# Do Different Mental Models Influence Cybersecurity Behavior? Evaluations via Statistical Reasoning Performance

**DOI:** 10.3389/fpsyg.2017.01929

**Published:** 2017-11-02

**Authors:** Gary L. Brase, Eugene Y. Vasserman, William Hsu

**Affiliations:** ^1^Department of Psychological Sciences, Kansas State University, Manhattan, KS, United States; ^2^Department of Computer Science, Kansas State University, Manhattan, KS, United States

**Keywords:** cybersecurity, mental models, Bayesian reasoning, human–computer interaction, metaphorical representation

## Abstract

Cybersecurity research often describes people as understanding internet security in terms of metaphorical mental models (e.g., disease risk, physical security risk, or criminal behavior risk). However, little research has directly evaluated if this is an accurate or productive framework. To assess this question, two experiments asked participants to respond to a statistical reasoning task framed in one of four different contexts (cybersecurity, plus the above alternative models). Each context was also presented using either percentages or natural frequencies, and these tasks were followed by a behavioral likelihood rating. As in previous research, consistent use of natural frequencies promoted correct Bayesian reasoning. There was little indication, however, that any of the alternative mental models generated consistently better understanding or reasoning over the actual cybersecurity context. There was some evidence that different models had some effects on patterns of responses, including the behavioral likelihood ratings, but these effects were small, as compared to the effect of the numerical format manipulation. This points to a need to improve the content of actual internet security warnings, rather than working to change the models users have of warnings.

## Introduction

Safety and security while using the internet is a serious concern for many people, and an entire area within computer science is devoted to dealing with the combination of cybersecurity and usability. One common example of a security issue is when users encounter an SSL/TLS (Secure Socket Layer/Transport Layer Security)^1^ warning as they attempt to visit a website. TLS warnings can occur as a result of server misconfigurations, self-signed certificates, or malicious activity (Fahl et al., 2014), so there are both fairly innocuous reasons and very legitimate security reasons for a user seeing this warning. The issue of how to increase understanding of, and compliance with, internet security warnings has been one of the hallmarks of cybersecurity research, and has necessitated some attention to the psychology of users: what do people understand about these warnings? How do users make decisions to proceed or not proceed to internet sites displaying such warnings?

Users’ responses to TLS warnings are relatively consistent: they usually ignore them (Akhawe and Porter-Felt, 2013; Porter-Felt et al., 2014). In general, previous studies have shown that users do not notice (or pay attention to) security indicators (Schechter et al., 2007), that users frequently ignore warnings that they do notice (Sunshine et al., 2009), and that users are generally oblivious to the possible implications of security warnings and indicators (Friedman et al., 2002; Wu et al., 2006; Egelman et al., 2008; Sunshine et al., 2009; Bravo-Lillo et al., 2013). More recent results have noted that users do pay some attention to phishing and malware warnings, whereas TLS warnings remain generally neglected. So in addition to the basic inherent security threats from ignored TLS warnings, there is now a danger that such warning will receive even less consideration from users because other warnings are perceived to be “more dangerous.” There does appear to be some malleability in the effectiveness of cybersecurity warnings. Different presentations of warnings (by different browsers and being used by different people) yield different results: only 33% of users click through the Mozilla Firefox TLS warnings, compared to 70% of Google Chrome users (Akhawe and Porter-Felt, 2013).

### Mental Models of Cybersecurity

Internet security, and indeed the entire internet, is a relatively novel context which does not have an established conceptualization. What type of conceptual frameworks do computer users bring to bear on these contexts? A prevalent approach to this topic is to assume that people understand cybersecurity through the use of particular metaphors, analogies, and mental models that are more familiar (Camp, 2006). To the extent that the metaphor used constitutes a strong overlap between the target domain and the original domain, it “works.” That is, the metaphor produces an understanding (or quasi-understanding) of the new topic based on a preexisting understanding of the established topic.

We can see a long string of efforts to understand and describe computer technology via metaphors. One notable early example is of computers running on smoke; when they break, all the smoke comes out.^2^ Another example is that the internet is a “series of tubes” which have a large but fixed and finite capacity. Delivery of data is slowed when the tubes are “filled” with too much material.^3^ More generally, a common metaphor for cyberspace is that of movement through physical space (“going to” a website, “surfing” the web, “visiting” a site, “following” a link, etc.). Given the ubiquity of metaphors used to describe computers and the internet, this raises a couple of compelling questions for people interested in cybersecurity issues. Within these presumed metaphorical conceptualizations, what corresponds to cybersecurity threats? How do these conceptualizations effect responses to those threats?

Researchers have identified users’ common underlying mental representation of TLS warnings, based on the idea that this representation is couched within a mental model of some other risk that is better understood. For example, users could internally model security risk as akin to a medical risk (viruses that attack the computer, etc.), or as akin to a theft risk (evil doers trying to steal money, property, or information), or as akin to a physical risk (computers that could “crash”). Camp (2006) proposed five possible models for communicating complex security risks. The models take the form of analogies or metaphors to other similar situations: physical security, medical risks/infections, crime/criminal behavior, warfare, and markets/economic failure. The idea is that these metaphors, or “folk models,” can be leveraged and adjusted to accomplish security goals of people designing computer systems to be safer for users (Wash, 2010; Bravo-Lillo et al., 2011). (Note that mental models held by laypersons differ significantly from those of experts; Asgharpour et al., 2007). The generic TLS warning does little to encourage or discourage these particular mental models, so users are relatively free to adopt whatever mental model they like. Which mental model a user uses, though, is proposed to affect their understanding, decisions, and behaviors:

(1) “Mental models can increase the predictive power of these agents when there are commonalities in the models used within a group, or when models used by one individual lead to a pattern of behavior across several tasks” (Blythe and Camp, 2012, p. 86).
(1) “Each different folk model leads users to make different choices when faced with these everyday computer security decisions” (Wash and Rader, 2015, p. 1).

It has not been established, however, if having insights into user’s mental models of cybersecurity is actually useful in terms of identifying or changing their related decisions and behaviors. That is, do different cybersecurity mental models actually carry meaningful implications in terms of insights or corrective actions, given the *ad hoc* nature of the models?

There is a substantial history of theorizing and research on the role of metaphors in human thinking (e.g., Lakoff and Johnson, 1980; Lakoff, 1987, 1993; Landau et al., 2014). A prevalent position within this literature, as exemplified by the above positions regarding cybersecurity, is that metaphors are important and key for if and how we understand much of the world around us. There are dissenting views on this issue (e.g., Murphy, 1996), but the idea of mental model metaphors as important factors has been dominant in the cybersecurity field.

The use of mental models in cybersecurity actually has two plausible interpretations, which are sometimes not distinguished but are important to clarify. A *strong intervention claim* is that mental models are necessary in order to understand the internet security situation (i.e., as a novel context, it can only be understood via a metaphor-based model). This strong claim, such as in the Blyth and Camp quote above, predicts that understanding and performance in cybersecurity situations is improved by the use of mental models; a metaphorical understanding will always be better than no understanding at all. A *weak intervention claim*, in contrast, is that mental models supplement understanding of the internet security situation. The weak claim, such as in the Wash and Rader quote above, predicts only that understanding and performance in cybersecurity situations will be *changed* by the use of mental models. Mental models will alter behavior, but not necessarily for the better. Thus, the weak claim implies some understanding of internet use and security on its own terms. To summarize, the strong intervention claim gives a strong rationale for the existence of mental models in this domain, but carries an implication that they must be fundamentally helpful (e.g., Blythe and Camp, 2012). A weak intervention claim does not imply that mental models must be helpful, but it also does not provide a justifying rationale for the use of those models in the first place (e.g., Wash and Rader, 2015).

### Current Research Design and Hypotheses

The purpose of the present research was to evaluate if, in fact, different mental models of cybersecurity situations can fundamentally change (and, by the strong intervention claim, improve) how people understand and reason about those situations. It has been proposed that some of the most common mental models for cybersecurity warnings (and other aspects of computers) are medical risk, criminal activity, and physical security. The two general hypotheses for this research are thus:

(1) Per the strong intervention view, understanding and performance in cybersecurity situations will be *improved* by relevant mental model metaphors, such as medical risk, criminal activity, and physical security (e.g., Blythe and Camp, 2012);
(1) Per the weak intervention view, understanding and performance in cybersecurity situations will be *altered* by relevant mental model metaphors, such as medical risk, criminal activity, and physical security (e.g., Wash and Rader, 2015).

A challenge, though, is that one cannot directly measure which mental model is active in a person’s mind. Indeed, the issue of what mental models exist in a person’s mind, and the extensiveness (i.e., “fleshing out”) of those mental models, is a basic ambiguity within the mental models framework (e.g., Rips, 1986, 1994; Bonatti, 1998; O’Brien et al., 1998).

Our solution to the question of which mental model is activated in the mind during the present studies is to explicitly invoke the domain of the mental model metaphor (and, for comparison, invoke the target domain of cybersecurity) within a computationally isomorphic context. The mental models theory implies that situations which clearly describe the different mental models should produce distinct and different patterns of understanding and reasoning even as the underlying structure of the situations are identical in all respects other than the framing.

The following two studies thus present people with situations that, while clearly invoking domains of different mental model metaphors, have isomorphic computational properties which people need to understand and reason about. Specifically, the elements of each situation can be arranged as pieces of information that can be used to calculate a posterior probability using Bayesian reasoning. (Bayesian reasoning involves combining base rate information with new information to obtain a revised estimate of the new rate; i.e., a posterior probability, based on the *a priori* probability and new evidence).

Why use Bayesian reasoning? Bayesian reasoning provides a *lingua franca* across the different mental model contexts and the actual context of internet security. It is an objective measure of how well people understand and think about these different situations. Bayesian reasoning also has a long history within psychology of being susceptible to various factors, producing – at different times – base rate neglect, accurate performance, and conservatism (overweighing the base rate). In short, Bayesian reasoning is generally a challenging task, which depends on a full understanding of the situation and clear thinking about the dynamics of the situation. Consistent with the thesis that different mental models might influence reasoning and decision making, Hafenbrädl and Hoffrage (2015) found that the contexts used in different Bayesian reasoning tasks (e.g., whether the cover story was about medical diagnosis, or predicting colored balls in an urn, or about college admission exams) actually could influence the responses of participants. Specifically, there were differences due to factors such as whether the task involved a norm violation or not and whether the stakes involved were high or low.

Lastly, research on Bayesian reasoning has also documented that the format of the numerical information within a task has a significant and reliable influence on performance. Specifically, presenting numbers as naturally sampled frequencies (a.k.a., natural frequencies) improves performance because it both simplifies the necessary calculations and provides a representation which most people find clearer and easier to understand (Gigerenzer and Hoffrage, 1995; Brase et al., 2006; Brase, 2009; Hill and Brase, 2012). The effect of different numerical formats provides a convenient baseline comparison effect; a reference to evaluate the validity of the task and any other observed effects. Interestingly, Hafenbrädl and Hoffrage (2015) noted that the cover story context effects in Bayesian reasoning (i.e., for norm violation and stakes involved) were more pronounced when the numerical information was presented using normalized numbers (e.g., percentages and single-event probabilities) relative to using natural frequencies.

In summary, there is ample evidence that the structure and content of Bayesian reasoning tasks influences people’s understanding and performance on those tasks. At the same time, it is a dimension of understanding and performance which can assessed consistently across different situations, including cybersecurity situations and the situations that have been proposed as the relevant mental model metaphors for understanding cybersecurity. The hypothesized effects of the strong and weak intervention claims for mental models of cybersecurity predict influences occurring in just these types of situations.

## Experiment 1

### Method

#### Participants

Data were collected from 274 undergraduate student participants at a large public university. The average age of the participants was 19.98, and the gender ratio was close to equal (157 females and 117 males). Per the researchers’ Institutional Review Board (IRB), all participants were given information about the study and consented to participate. By electing to participate in this study, these participants received credit toward partial fulfillment of their introductory psychology course. Two participants completed the primary task for this study but failed to answer the follow-up questions, and they were therefore excluded from those analyses. (This level of data exclusion due to human participants failing to complete tasks is quite low, compared to similar judgment and decision making research).

#### Materials and Procedures

Participants completed an online collection of tasks and surveys, which included variations of a Bayesian reasoning task, each of which was presented to roughly an equal number of participants in a between-subjects, 2 × 4 design. All the Bayesian reasoning task variants had the same basic form:

(1) A context was described in which a key event sometimes occurs, predicted by a certain cue, noting that there is some variation in the occurrence of event and the cue. Specific information was then given about how often the event occurs and how it relates to the cue.
(1) The specific data (which was identical in every context) was that there was a 2% [2 out of every 100] base rate of an event occurring overall, and these events all occurred in the presence of the cue. Furthermore, the false positive rate (the cue sometimes occurs absent the event) was 8.1% [8 out of 98].
(1) Participants were then asked to judge the probability of the event, given the occurrence of the cue (i.e., the posterior probability).

This basic form was developed within one of four different context stories; a cybersecurity context and three different mental models which have been proposed for understanding cybersecurity contexts:

(1) Cybersecurity context (the likelihood of unsafe websites, given a security warning).
(1) Disease context (the likelihood of sick people, given coughing and sneezing).
(1) Physical security context (the likelihood of physical assaults, given a poorly lighted street).
(1) Crime/criminal behavior context (the likelihood of credit card fraud, given a suspicious activity alert).

Each of these four Bayesian reasoning context stories was presented in one of two different numerical formats: percentages and natural frequencies. The Supplementary Material provides the full texts of the stimuli for all the conditions. After the Bayesian reasoning task, all participants were asked a follow-up question of the following form (with details consistent with their Bayesian reasoning task filled in): “Think about when you are [in the previously described context]. You have [engaged in the base rate activity], but then [risk cue occurs]. What do you do?” Participants were given a 1–7 scale to answer this question, with 1 labeled as heeding the cue and 7 labeled as ignoring the cue. Due to the online nature of this study, participants were able to take as much time as they wanted in completing the tasks. Upon completion of all the tasks, participants were provided with debriefing information about the research and their data were stored with only an identification code, ensuring anonymity.

### Results

**FIGURE 1:**
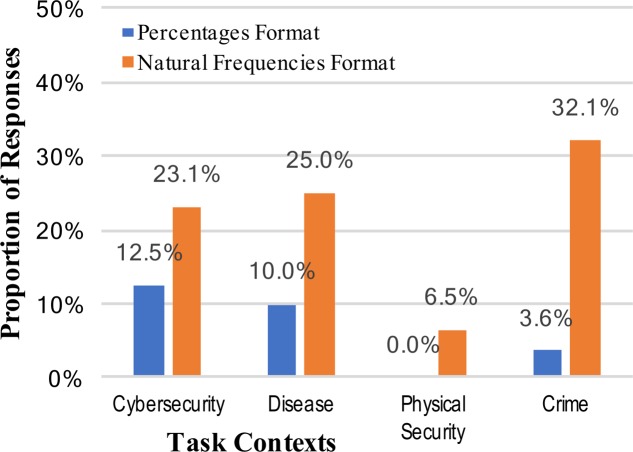
Percentages of participants who reached the correct posterior probability answer to the Bayesian reasoning task, across the context stories and numerical format conditions.

Answers were scored as correct only if they were the exact correct answer, but were accepted as correct in any format (e.g., 2/10, 0.20, or 20%). As expected, Bayesian inference was a difficult task for participants, but providing information in the form of natural frequencies significantly improved performance (7.4% vs. 21.7%, collapsed across context stories, using a difference of proportions test; Blalock, 1979): *z* = 3.37, *p* = 0.0004, and *h* = 0.42 (see **Figure 1**). There was little evidence for the strong intervention hypothesis, however, that any particular mental model or metaphorical framing of the context improved people’s understanding of internet security situations. In fact, the cybersecurity context (not employing any mental model metaphor) elicited the best overall performance when looking across both numerical formats, whereas the physical assault context elicited significantly worse performance than any other context (vs. cybersecurity context: *z* = 2.60, *p* = 0.005, and *h* = 0.50). If anything, the results suggest that the physical assault mental model *inhibited* accurate situational understanding and reasoning. We are forced to conclude that some of the most commonly used mental models for cybersecurity (disease risk, physical assault, criminal behavior) do not significantly improve performance relative to a version of the task actually describing a literal cybersecurity situation, contrary to the predictions of the strong intervention view.

**FIGURE 2:**
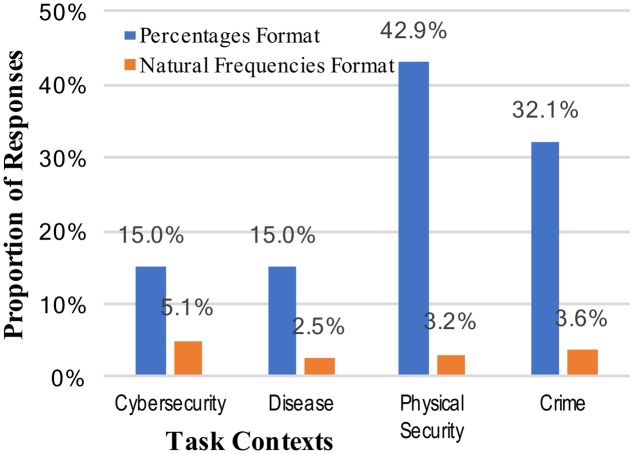
Percentages of participants who incorrectly answered the Bayesian reasoning task with the base-rate (conservatism), across the context stories and numerical format conditions.

The weak intervention hypothesis predicts systematic changes in performance across different mental models, although not necessarily improved performance. The varying levels of correct performance across contexts may support this view, and further evidence can be brought to bear on this prediction by looking at other common (incorrect) responses. Two of the most common erroneous responses in Bayesian reasoning situations are not attending to the base rate at all (base rate neglect) or relying too much on the base rate (conservatism). These correspond, respectively, to the specific answers of either 0.919 (91.9%; the inverse of the cue false positive rate [92% was accepted as a correct, rounded answer]), or 0.02 (2%; the base rate). Scoring of answers was again done, and base rate neglect was observed for only two participants across all the conditions of the study. Reporting of the actual cue false positive rate, failing to invert it, was observed only once. The consistent absence of this type of answer is unsupportive of the weak intervention view, but an alternative reason for these results is that the tasks were simply too difficult to elicit consistent answers (although note that rates of correct responses in **Figure 1**). The rates of conservatism –the answer of the original base rate without the new information incorporated – are shown in **Figure 2**.

The rates of conservatism, like the correct answers, were significantly different for different numerical formats. Specifically, answering with the base rate very rarely occurred with natural frequencies (24.3% vs. 3.6%, collapsed across context stories: *z* = 4.96, *p* < 0.001, and *h* = 0.65; see **Figure 2**). There was some limited evidence of different mental models changing the patterns of answers; i.e., when information was given in percentages the rates of conservatism varied from 15 to 43% (a significant change: *z* = 2.57, *p* = 0.006, and *h* = 0.63). The rates of conservative responses when the information was given in natural frequencies, however, was very consistently low.

A comparison of **Figures 1**, **2** suggests an approximate symmetry: the rates of base rate conservation are roughly inverses of the correct response rates. This might be the best support for a weak intervention account of mental models. At the same time, however, the much larger effect apparent across all these results is that of numerical format.

We now consider tendencies to heed or ignore the warning cue across these tasks. Perhaps mental models do not make any major difference in understanding or reasoning about cybersecurity situations, but particular models could have effects in terms of making people more attentive to the risks of unsafe internet behaviors. The ratings of behavioral likelihood following the Bayesian task were evaluated using a 2 × 4 factorial analysis of variance (ANOVA; Fisher, 1925), which found significant differences across context stories [*F*(3,264) = 20.267, *p* < 0.001, $\eta_{p}^{2}$ = 0.187]. *Post hoc* analyses indicate that this significant effect is driven by the crime/criminal behavior context being rated significantly lower than other contexts (i.e., more heeding of the risk cue), and the disease context being rated as significantly higher than other contexts (i.e., more ignoring of the risk cue; see **Table 1**). Thus, physical assault and disease mental models do not improve desired perceptions and responses to cybersecurity risks. Only a criminal behavior mental model may have some utility in this regard.

**Table 1:** Participants’ mean ratings of how likely they would be to heed (1) or ignore (7) a cue within each context story and given different numerical formats (with standard deviations given in parentheses).

	Percentages format	Natural frequencies format
Cybersecurity context	3.5 (±1.8)	3.0 (±1.8)
Disease context	4.6 (±1.3)	4.6 (±1.5)
Physical security context	3.6 (±1.6)	3.9 (±1.8)
Crime/criminal behavior context	2.6 (±1.6)	2.4 (±1.1)


The remaining results from the ANOVA were that the likelihood ratings were completely unaffected by the numerical format used in the task [*F*(1,264) = 0.389, *p* = 0.533] and there was no interaction between the context story and numerical format [*F*(3,264) = 0.743, *p* = 0.528]. The only other notable result from the analysis was that the likelihood ratings had unequal variance, with the cybersecurity context producing more variable responses than other contexts [*F*(7,264) = 2.661, *p* = 0.011].

## Experiment 2

One might argue that Experiment 1 involved reasoning about four fundamentally different situations, and therefore mental models (or metaphors) were not actually involved. Or, along a similar line of thought, that the different contexts were somehow not sufficiently related to the target context of cybersecurity. Lastly, there could be a concern that the results of Experiment 1 were somehow due to the particular numbers that were used (i.e., a 2% base-rate and 8.1% false alarm rate) or that the numerical format manipulation somehow inhibited mental model effects.

Experiment 2 therefore involves a statistical reasoning task that de-emphasizes the numerical format differences and Bayesian reasoning aspects of the task while also explicitly embedding the model metaphors within a cybersecurity context. The results of this experiment thus focus on differences in performance generally (as opposed to correct Bayesian inferences) and utilizes a consistent question format at end of all task conditions (even as that creates issues for Bayesian reasoning performance). These changes will allow further evaluations of the strong and weak intervention hypotheses described previously.

### Method

#### Participants

A total of 244 undergraduate students at a large public university were recruited as participants in a study that was composed of several unrelated tasks, and 234 participants actually provided responses to the task in this experiment. The following data and analyses therefore refer to this sample. The average age of the participants was 19.70, and the gender ratio was close to equal (108 males and 126 females). These participants participated under the same protocol as in Experiment 1 with regards to informed consent, receiving credit toward partial fulfillment of their introductory psychology course, data anonymization, and debriefing procedure.

#### Materials and Procedures

Participants completed an online collection of tasks and surveys, which included variations of a Bayesian reasoning task, each of which was presented to roughly an equal number of participants in a between-subjects, 2 × 4 design. All the Bayesian reasoning task variants had the same basic form, as described in the previous experiment. In contrast to the task in Experiment 1, however, the specific numbers used were changed to simplify the task (a 1% base rate and a 1% false alarm rate), and the contexts given were varied in a different manner (see full texts in the Supplementary Material):

(1) A control condition context described a cybersecurity context, using no additional mental models as metaphors.
(1) A Cybersecurity context + Disease model condition described a cybersecurity context and also recommended a metaphor of the disease mental model.
(1) Cybersecurity context + Physical security model condition described a cybersecurity context and also recommended a metaphor of the physical security mental model.
(1) Cybersecurity context + Crime/criminal behavior model condition described a cybersecurity context and also recommended a metaphor of the crime/criminal behavior mental model.

Each of these contexts was presented with numbers either in percentages or natural frequencies, as in Experiment 1. All the conditions, regardless of how the initial numerical information was given, had the same questions at the conclusion of the information given in context:

(1) Out of 100 websites, how many will generate security warnings, either because they are truly unsafe or by mistake? [i.e., asking for the total positive rate; this is a new items, relative to Experiment 1].
(1) Overall, what is the likelihood that a security warning is for a truly unsafe website? [i.e., asking for the posterior probability, in a non-committal numerical format].
(1) Think about when you are on the Internet and visiting websites. You have visited a large number of sites, all is going well, but then a security warning pops up to tell you that the site you are trying to go to is unsafe. What do you do? (rated on a 1–7 scale to answer this question, with 1 labeled as heeding the cue and 7 labeled as ignoring the cue, just as in Experiment 1).

Note that, because the posterior probability question used here (unlike in Experiment 1) is identical for both the percentage and natural frequency numerical format conditions, we can anticipate that his will hamper the performance facilitation effects for naturally sampled frequencies. The Supplementary Material provides the full texts of the stimuli for all the conditions.

### Results

**FIGURE 3:**
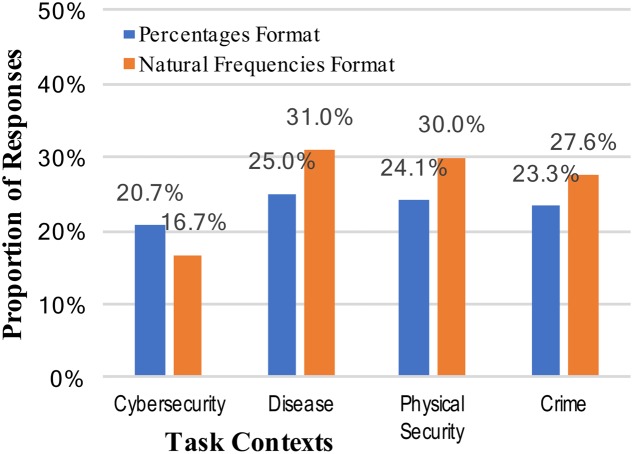
Percentages of participants who reached the correct positive test rate (2 out of 100), across the context stories and numerical format conditions.

Answers for the total positive rate were considered correct for any answer of “2,” regardless of format (numeral or written out “two”). There was a very small, non-significant increase in correct responses for the natural frequency format over the percentage format (23.3% vs. 26.3%, collapsed across context stories; see **Figure 3**): *z* = 0.53, *p* = 0.298, and *h* = 0.07.

**FIGURE 4:**
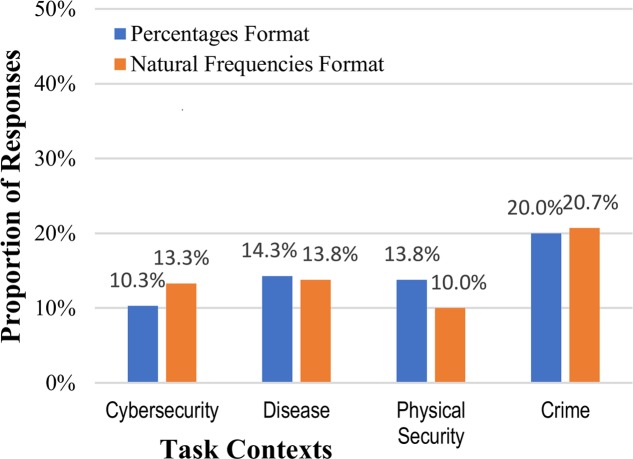
Percentages of participants who reached the correct posterior probability answer to the Bayesian reasoning task (1 out of 2, or 50%), across the context stories and numerical format conditions.

Answers for the posterior probability were considered correct for any answer of 0.5, regardless of format (0.5, 50%, ½, or 1 out of 2). Because this question asked for an answer in a very open-ended manner, “what is the likelihood…” there was more variation in responses, including several answers such as “not likely” and “very likely” (these were not considered correct). As in Experiment 1, calculating the posterior probability was difficult for participants, and because of the very general phrasing of the question there as not a significant effect of numerical format on performance (14.6% vs. 14.5%, collapsed across context stories): *z* = 0.02, *p* = 0.491, and *h* = 0.003 (see **Figure 4**). Inconsistent with either the strong or weak intervention hypotheses, none of the different mental models of the context significantly changed people’s reasoning about the internet security situation.

**FIGURE 5:**
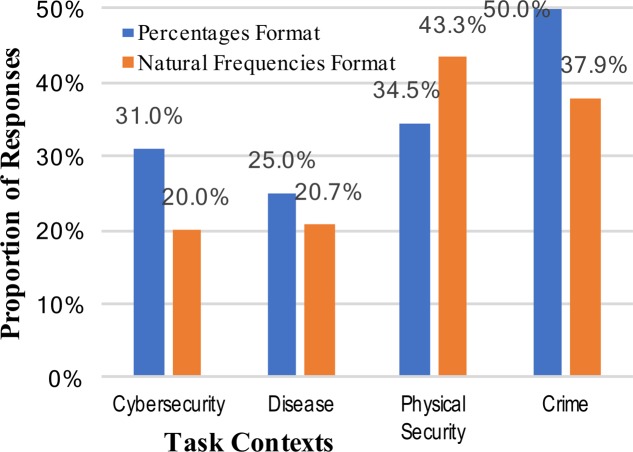
Percentages of participants who incorrectly answered the Bayesian reasoning task with the base-rate / total positive rate (1 or 1%), across the context stories and numerical format conditions.

To further evaluate any effects on performance across different mental models for cybersecurity, we again looked at common incorrect responses. The rates of conservatism (relying too much on the base rate) and base rate neglect (not attending to the base rate) could produce similar responses in this task, particularly given the open-ended nature of the question. All responses which could be interpreted as these types of responses (1, 1%, 0.01, etc.) were therefore considered as errors of these type. **Figure 5** shows that these were, cumulatively, fairly common responses, unlike in Experiment 1. As with the correct posterior probability answers, there was a slight difference across different numerical formats, with this particular wrong answer more prevalent for the percentage format, but it was again not significant (35.1% vs. 30.5%, collapsed across context stories: *z* = 0.75, *p* = 0.227, *h* = 0.098). Adding a metaphorical mental model to the cybersecurity context, if anything, increased the likelihood of these particular types of incorrect answers (**Figure 5**). This result is therefore inconsistent with the strong intervention hypothesis, but possibly consistent with the weak intervention hypothesis.

Turning to the rated tendencies to heed or ignore the warning cue across these tasks (**Table 2**), a 2 × 4 factorial ANOVA found no significant differences across context stories [*F*(3,226) = 0.663, *p* = 0.576, $\eta_{p}^{2}$ = 0.009] or across numerical format [*F*(1,226) = 0.024, *p* = 0.877, $\eta_{p}^{2}$ < 0.001], and no interaction [*F*(3,226) = 0.346, *p* = 0.792, $\eta_{p}^{2}$ = 0.005]. Thus, Experiment 2 was unable to replicate the small effect of a criminal behavior mental model on desired perceptions and responses to cybersecurity risks.

**Table 2:** Participants’ mean ratings of how likely they would be to heed (1) or ignore (7) a cue within each context story and given different numerical formats (with standard deviations given in parentheses).

	Percentages format	Natural frequencies format
Cybersecurity (no additional model)	3.6 (±1.8)	3.8 (±1.9)
Cybersecurity + disease model	3.6 (±2.2)	3.2 (±2.1)
Cybersecurity + physical security model	3.4 (±1.9)	3.2 (±1.8)
Cybersecurity + Crime model	3.2 (±1.6)	3.3 (±1.7)


## Conclusion

The present study compared the effects of several different mental models commonly thought to be used by people to understand the nature of the internet and threats that exist on the internet (i.e., a disease mental model, a physical security mental model, and a crime/criminal behavior mental model). The results indicate that users’ understanding of these contexts, how they reason about these contexts, and their projected behaviors within these contexts are very similar in many ways. Specifically, the process of Bayesian reasoning (determining the posterior probability of an event, given a cue) is similar across these contexts: a known manipulation that influences Bayesian reasoning, the numerical format of the information provided, led to an effect across all these mental models. However, the quality of Bayesian reasoning performance was not significantly better with any of these mental models than it was with the actual cybersecurity context. The implication is that the tested mental models do not have an appreciable (positive) effect on how users reason about these situations. Follow-up questions found that the Bayesian reasoning context story was also not related to their rated likelihood of heeding or ignoring the event-predictive cue. These results are inconsistent with the strong intervention hypothesis, which predicts that the use of mental model metaphors will facilitate understanding and performance in the target context (e.g., Camp, 2006; Blythe and Camp, 2012). There are some results which fit with the weak intervention hypothesis (i.e., certain incorrect answers may be more or less common when different mental model metaphors are employed; e.g., Wash and Rader, 2015), but that evidence is inconsistent.

The weak intervention hypothesis is, of course, not orthogonal to the strong intervention hypothesis in this research. Specifically, the strong intervention prediction of improved performance when using mental models is a conceptual subset of the effects predicted by the weak intervention prediction of altered performance generally. One could possibly create a study in which the results could in principle support the strong intervention claim and counter the weak intervention claim (e.g., repeated evidence of only improvements across several tasks that employed mental models), but that direction of research does not appear to be necessary given the present results.

It is not plausible that the current failure to find improved understanding and reasoning while using the various mental models is due to systemic methodological issues or low statistical power, because we concurrently replicated the effect of using natural frequencies vs. percentages (Experiment 1). It is also not the case that particular numerical structures or posterior probability question formats were problematic because Experiment 2, with simpler numbers and a uniform question format, still found no effects of mental models. On the other hand, Experiment 2 clearly and explicitly promoted the use of different mental models of cybersecurity across different conditions (including a control condition of no promoted model).

The use of Bayesian reasoning as a “sandbox” for evaluating mental models of cybersecurity is not the only possible methodology one could use for this type of research. There are a number of other “sandboxes,” if you will, which can be used as methodologies for evaluating mental models of cybersecurity and to access understanding and performance. Bayesian reasoning is, however, a particularly well-suited arena for this type of work: one can view many cybersecurity issues as a general belief-updating situation, in which a person has a priori beliefs about their internet security and they need to revise those beliefs in light of new information (i.e., do Bayesian reasoning). One might argue that the effects of different mental models on understanding and behavior in cybersecurity situations are more subtle than the present research is able to detect, but this raises a further question of whether such small or fickle effects are sufficiently important for cybersecurity research. Would they even cause significant behavioral deviation if their cognitive counterparts could be detected? One may alternatively argue that the stakes differ by context (e.g., catching a cold vs. physical assault), and that this can influence Bayesian reasoning (Hafenbrädl and Hoffrage, 2015), so therefore these influences must be controlled for in order to evaluate any effects of the contexts a mental model metaphors. The difficulty with this argument is that it is essentially about the partialing of effects as due to different factors, but these factors have not been identified in any principled way (e.g., other than *post hoc*).

There are also several further studies which can extend the present research and assess its generalizability. For example, to what extent are Bayesian reasoning performances predictive of actual behaviors? (In other literatures this connection is well-established, e.g., in the context of HIV tests, see Gigerenzer et al., 1998, but does it apply to TLS warning responses?). It is also possible that there could be unanticipated changes in people’s responses if one were to factor in prior knowledge or behavioral tendencies regarding TLS warnings, general computer literacy, pre-existing preferences for certain mental models, Finally, the present results are based on self-reports of likely behaviors, and it is possible that actual behaviors (within cybersecurity or in the analogous contexts) could differ to some extent in real-world situations. It is possible that people are more vigilant or cautious in their actual behaviors, although it seems at least as likely (given typical gaps between intentions and behaviors; Sheeran, 2002; Sutton, 2006) that people are even less responsive to risks in actual situations. More ecologically realistic studies could address this concern.

### Practical Applications

Collectively these results cast into doubt the idea that using metaphorical mental models to think about cybersecurity has utility for internet users or cybersecurity research. The results suggest that peoples’ thinking instead can be understood generally (across all the different mental models) and more directly in terms of how well they understand the risks, rewards, and signals they receive in their environment.

From this view, greater focus should be trained on the format and content of security warnings that are given to internet users. This focus leads to an adjusted set of questions with regard to how one can improve cybersecurity behaviors such as bypassing TLS warnings (Akhawe and Porter-Felt, 2013; Porter-Felt et al., 2014). Do users correctly and fully understand these warnings? Do the warnings reliably and validly signal security threats (both in reality and in the judgments of users)? It may be more productive to fundamentally change the way internet security threats are communicated to users, than to work on the mental models users might impose on those communications. For example, the security system could bypass giving a direct warning to the user, but rather redirect them to a known safe site. Such a default “opt in” system might then unobtrusively inform users regarding why they were redirected and give them a chance to reverse the safety decision already made on their behalf. This could not only improve safer online behavior, but it could also encourage a view of security warnings as more effectively protecting users (i.e., perceptions of reliability and validity).

None of this is to deny that people often have metaphorical understandings (i.e., mental models) of internet situations. Like a vast range of other contexts in life, people use metaphors to think about situations (Pinker, 2000, 2007). Focusing on the models rather than the actual situation, though, does not appear to strongly predict understanding, reasoning, or likely behaviors within the actual situation.

## Ethics Statement

This study was carried out in accordance with the recommendations of the Kansas State University Institutional Review Board (KSU IRB), with written informed consent from all subjects. All subjects gave written informed consent in accordance with the Declaration of Helsinki. The protocol was approved by the KSU IRB.

## Author Contributions

GB was the lead researcher on this project, with EV and WH contributing at all stages of the research process.

## Conflict of Interest Statement

The authors declare that the research was conducted in the absence of any commercial or financial relationships that could be construed as a potential conflict of interest.

## Footnotes

1 For ease of reference, we will refer to SSL/TLS as just TLS for the remainder of this paper.

2 http://www.catb.org/jargon/html/M/magic-smoke.html

3 http://www.washingtonpost.com/blogs/wonkblog/post/the-internet-is-in-fact-a-series-of-tubes/2011/09/20/gIQALZwfiK_blog.html
