# Supplementary material for: Do Different Mental Models Influence Cybersecurity Behavior? Evaluations via Statistical Reasoning Performance
Source: Front Psychol. 2017 Nov 2;8:1929. doi: 10.3389/fpsyg.2017.01929 (PMC5673648; doi:10.3389/fpsyg.2017.01929)
Supplement: Supplementary file 1 [file Presentation_1.PDF]

Supplementary Material

How do people understand Cybersecurity? Statistical reasoning performance shows no benefit to different mental model representations

Gary L. Brase*, Eugene Y. Vasserman, William Hsu

*** Correspondence:** Gary L. Brase: gbrase@ksu.edu

1 Supplementary Material: Experiment Stimuli

For all stimuli the percentage format of the conditions is shown initially, with the naturally sampled frequency format shown in brackets.

Experiment 1: Cybersecurity context

Most individuals go to numerous websites every day. Individuals also have some general idea that there are some unsafe websites (for example, sites that try to steal personal information, infect computers with viruses, and hijack personal computers to help conduct various illegal activities). To help protect against these hazards, computers have security programs: firewalls, anti-virus programs, and other safety features. So individuals will often notice that, as they surf the web, they occasionally will get security warnings when they try to go to a particular website. Here is some specific information about these unsafe websites and the security warnings they generate.

A recent survey found that 2% of [2 out of every 100] websites were unsafe. These unsafe websites produced security warnings. Sometimes security warnings are also produced, however, by other websites even though they were not critically unsafe. Specifically, 8.1% of [8 of the 98] safe websites also generated security warnings.

Consider your own use of websites. You can expect to, eventually, encounter a security warning. [You can expect to, eventually, visit 100 websites over some period of time. Of those 100 websites, how many security warning would you expect to get and how many of those warnings will be for a truly unsafe website?]

When you get a security warnings, what is the probability that it is a truly unsafe website? _____

[Of 100 websites, _____ will generate security warnings, and _____ of these will be for truly unsafe websites.]

[Followed by]

Think about when you are on the Internet, visiting websites. You have visited a large number of sites, all is going well, but then a security warning pops up to tell you that the site you are trying to go to is unsafe. What do you do? (rated on a 1-7 scale, in which 1 was labeled as "Definitely **heed** the warning and do not go to this website" and 7 was labeled as "Definitely **ignore** the warning and proceed to the website.")

Experiment 1: Disease Context

Most individuals interact with numerous other people every day. Individuals also have some general idea that there some sick people out in the world (for example, people with colds, influenza, and other viral infections). To help protect against these sicknesses, individuals can avoid physical contact with people who show symptoms of being sick, such as coughing and sneezing. So individuals will often notice that, as they go about interacting with people, they occasionally will meet someone who coughs and sneezes. Here is some specific information about when people are sick and when they cough or sneeze.

A recent survey found that 2% of [2 out of every 100] people were sick. These sick people showed either coughing or sneezing (or both). Sometimes coughing or sneezing also occur, though, in perfectly healthy people. Specifically, 8.1% of the healthy people in the survey also happened to cough or sneeze [while interacting with 8 of the 98 healthy people in the survey, they too happened to cough or sneeze].

Consider your own interactions with other people. You can expect to, eventually, meet someone who is coughing or sneezing [100 other people over some period of time. Of those 100 people, how many times would you expect to encounter someone who is coughing or sneezing, and how many of those coughing/sneezing episodes would be indications of having met with a truly sick person?]

When you meet someone who is coughing or sneezing, what is the probability that this person is truly sick? _____

[*Of 100 people met,* _____ *will be coughing or sneezing, and* _____ *of these will be because that person was actually sick*]

[Followed by]

Think about when you are out in public, meeting people. You have met a large number of people, all is going well, but then you meet someone who is sneezing/coughing. What do you do? (rated on a 1-7 scale, in which 1 was labeled as "Definitely **heed** the warning and walk away from this person" and 7 was labeled as "Definitely **ignore** the warning and keep talking with this person.")

Experiment 1: Physical security context

Many people walk on city streets at night as they go about their business. City streets, however, can be unsafe at night because of muggers and others who might physically assault them. To help protect against these hazards, experts recommend taking precautionary

measures such as avoiding areas with poor lighting. Most people pay attention to these recommendations when they walk around in the city, but not always.

Here is some specific information about how often physical assaults occur on city streets at night and how they are related to the street lighting.

A recent survey found that 2% of [2 out of every 100] people walking on city streets at night were physically assaulted. These assaults all occurred in places with poor lighting. Sometimes city streets were poorly lit, however, yet people who walked through that area were not assaulted at all. Specifically, 8.1% of [8 of the 98] perfectly safe city streets also were poorly lit.

Consider your own experiences with walking down city streets at night. You can expect to, eventually, encounter a poorly lit street [walk down a city street 100 times].

When you walk down a dark city street, what is the probability that you will be physically assaulted? _____

[*Of those 100 walks,* _____ *will be down dark streets, and* _____ *of these will lead to being physically assaulted.*]

[Followed by]

Think about when you are in a city, walking down its streets. You have walked down a number of streets, all is going well, but then you come across a poorly lit section of road that is directly in the path to your destination. What do you do? (rated on a 1-7 scale, in which 1 was labeled as “Definitely **heed** the fact that the street is poorly lit and walk in a different direction” and 7 was labeled as “Definitely **ignore** the fact that the street is poorly lit and walk down it.”)

Experiment 1: Crime/criminal behavior

Many people use credit cards. People also have some general idea that there are some criminals who steal credit card information (for example, intercepting the card number at a store, at an ATM, or somewhere else). To help protect against these card number thefts and fraud, many credit card companies will contact card owners if there is suspicious card activities on their account. So individuals will sometimes notice that, as they go about buying things with their credit card, they occasionally may get a call from their credit card company asking if they are making the purchases showing up on the card.

Here is some specific information about credit card purchases, credit card fraud, and the “suspicious activity” phone calls they generate.

A recent survey found that 2% of [2 out of every 100] credit card purchases were instances of credit card fraud. These purchases with stolen credit cards produced suspicious activity alerts. Sometimes suspicious activity alerts were also produced, however, by legitimate purchases of the actual card owner. Specifically, 8.1% of [8 of the 98] legitimate purchases also generated suspicious activity alerts.

Consider your own use of credit cards (or imagine you have a credit card). You can expect to, eventually, get a suspicious activity alert [have 100 purchases on your credit card record over some period of time].

When you get a suspicious activity alert, what is the probability that it will be for actual credit card fraud?

[Over the course of th ose 100 listed purchases, how many suspicious activity alerts would you expect to get and how many of those alerts will be for actual credit card fraud?]

Of 100 listed purchases, _____ will generate suspicious activity alerts, and _____ of these will be for actual credit card fraud.

[Followed by]

Think about when you are using a credit card to make purchases. You have made a number of purchases with this credit card in the past, and it has not been a problem, but then you get a phone call from your credit card company about suspicious activity on your credit card. What do you do? (rated on a 1-7 scale, in which 1 was labeled as “Definitely **heed** the alert and cancel this credit card” and 7 was labeled as “Definitely **ignore** the alert and continue to use this credit card.”)

Experiment 2: Cybersecurity (no additional model)

Most individuals go to numerous websites every day. People also have a general idea that there are some unsafe websites (for example, sites that try to steal personal information, infect computers with viruses, and hijack personal computers to help conduct various illegal activities). To help protect against these hazards, computers have security programs: firewalls, anti-virus programs, and other safety features. So individuals will often notice that, as they surf the web, they occasionally will get security warnings when they try to go to a particular website. Here is some specific information about these unsafe websites and the security warnings they generate:A recent survey found that 1% of websites [1 out of every 100 websites] were unsafe. These unsafe websites produced security warnings. Sometimes security warnings are also produced, however, by other websites even though they were not critically unsafe. Specifically, 1% of [1 of the 99] safe websites also generated security warnings. Consider your own use of websites. You can expect to, eventually, encounter a security warning [visit a large number of websites over some period of time].

Experiment 2: Cybersecurity + Disease Model

Most individuals go to numerous websites every day. People also have a general idea that there are some unsafe websites (for example, sites that try to steal personal information, infect computers with viruses, and hijack personal computers to help conduct various illegal activities). To help protect against these hazards, computers have security programs: firewalls, anti-virus programs, and other safety features. So individuals will often notice that, as they surf the web, they occasionally will get security warnings when they try to go to a particular website. Computer security programs work something like the way a doctor can use a symptom (such as a fever) as an indication that a patient has an illness. A symptom is not a perfect indication, of course – people can have a fever for a variety of reasons, but it is a warning sign. Here is some specific information about these unsafe websites and the security warnings they generate. A recent survey found that 1% of websites [1 out of every 100 websites] were unsafe. These unsafe websites produced security warnings. Just like fever indicating an illness, certain website features trigger security warnings. However, sometimes security warnings are also produced by other websites even though they were not critically unsafe (like a fever for some reason other than illness). Specifically, 1% of [1 of the 99] safe websites also generated security warnings. Consider your own use of websites and, metaphorically, your experiences a fever as a symptom of being sick. You can expect to, eventually, encounter a security warning, much like you will eventually have a fever at some point [visit a large number of websites over some period of time].

Experiment 2: Cybersecurity + Physical Security Model

Most individuals go to numerous websites every day. People also have a general idea that there are some unsafe websites (for example, sites that try to steal personal information, infect computers with viruses, and hijack personal computers to help conduct various illegal activities). To help protect against these hazards, computers have security programs: firewalls, anti-virus programs, and other safety features. So individuals will often notice that, as they surf the web, they occasionally will get security warnings when they try to go to a particular website. Computer security programs work something like being alerted when a situation could potentially be physically risky (for example walking down a city streets at night). Certain features, like poor street lighting, can be used to indicate when the situation is

too risky. Poor street lighting is not a perfect indication, of course – a poorly lit street is not always dangerous, but it is a warning sign. Here is some specific information about these unsafe websites and the security warnings they generate. A recent survey found that 1% of websites [1 out of every 100 websites] were unsafe. These unsafe websites produced security warnings. Just like poor lighting indicates a physically risky location, certain website features trigger security warnings. However, sometimes security warnings are also produced by other websites even though they were not critically unsafe (like poor lighting in a place that is not actually risky). Specifically, 1% of [1 of the 99] safe websites also generated security warnings. Consider your own use of websites and, metaphorically, your experiences with cues (like poor lighting) of physically dangerous situations. You can expect to, eventually, encounter a security warning, much like you will eventually come across a poorly lit street at some point [visit a large number of websites over some period of time].

Experiment 2: Cybersecurity + Crime Model

Most individuals go to numerous websites every day. People also have a general idea that there are some unsafe websites (for example, sites that try to steal personal information, infect computers with viruses, and hijack personal computers to help conduct various illegal activities). To help protect against these hazards, computers have security programs: firewalls, anti-virus programs, and other safety features. So individuals will often notice that, as they surf the web, they occasionally will get security warnings when they try to go to a particular website. Computer security programs work something like the way credit card companies monitor accounts for suspicious card activities, as an indication that there might be some sort of credit card theft or fraud going on. A suspicious credit card activity is not a perfect indication, of course – actual credit card owners could be legitimately using their own card for various things that get flagged as “suspicious”, but it is a warning sign. Here is some specific information about these unsafe websites and the security warnings they generate. A recent survey found that 1% of websites [1 out of every 100 websites] were unsafe. These unsafe websites produced security warnings. Just like suspicious activity indicating credit card fraud, certain website features trigger security warnings. However, sometimes security warnings are also produced by other websites even though they were not critically unsafe (like suspicious looking activity for some reason other than fraud). Specifically, 1% of [1 of the 99] safe websites also generated security warnings. Consider your own use of websites and, metaphorically, your experiences with credit cards and credit card security warnings. You can expect to, eventually, encounter a security warning, much like you will eventually get a suspicious activity warning for your credit card at some point [visit a large number of websites over some period of time].

All of the above conditions for Experiment 2 included the following three questions after the different context information, in this same wording:

Out of 100 websites, how many will generate security warnings, either because they are truly unsafe or by mistake? (1)

Overall, what is the likelihood that a security warning is for a truly unsafe website? (2)

Think about when you are on the Internet and visiting websites. You have visited a large number of sites, all is going well, but then a security warning pops up to tell you that the site you are trying to go to is unsafe. What do you do?

- ☐ Definitely heed the warning and do not go to this website (1)
- ☐ (2)
- ☐ (3)
- ☐ (4)
- ☐ (5)
- ☐ (6)
- ☐ Definitely ignore the warning and proceed to the website (7)
